# Genome-Wide Identification of Barley Long Noncoding RNAs and Analysis of Their Regulatory Interactions during Shoot and Grain Development

**DOI:** 10.3390/ijms22105087

**Published:** 2021-05-11

**Authors:** Sebastian Gasparis, Mateusz Przyborowski, Anna Nadolska-Orczyk

**Affiliations:** Department of Functional Genomics, Plant Breeding and Acclimatization Institute—National Research Institute, 05-870 Błonie, Radzików, Poland; m.przyborowski@ihar.edu.pl (M.P.); a.orczyk@ihar.edu.pl (A.N.-O.)

**Keywords:** lncRNA, barley, RNAseq, grain development, shoot development

## Abstract

Long noncoding RNAs (lncRNAs) are a class of RNA molecules with gene regulatory functions in plant development and the stress response. Although the number of lncRNAs identified in plants is rapidly increasing, very little is known about their role in barley development. In this study, we performed global identification of barley lncRNAs based on 53 RNAseq libraries derived from nine different barley tissues and organs. In total, 17,250 lncRNAs derived from 10,883 loci were identified, including 8954 novel lncRNAs. Differential expression of lncRNAs was observed in the developing shoot apices and grains, the two organs that have a direct influence on the final yield. The regulatory interaction of differentially expressed lncRNAs with the potential target genes was evaluated. We identified 176 cis-acting lncRNAs in shoot apices and 424 in grains, while the number of trans-acting lncRNAs in these organs was 1736 and 540, respectively. The potential target protein-coding genes were identified, and their biological function was annotated using MapMan ontology. This is the first insight into the roles of lncRNAs in barley development on the genome-wide scale, and our results provide a solid background for future functional studies.

## 1. Introduction

In eukaryotic organisms, more than 90% of the genome is transcribed, but only a small of its fraction (2–25%) encodes proteins [1,2]. The remaining part is transcribed to different classes of noncoding RNAs (ncRNAs), which for decades have been recognized as transcriptional noise without any biological importance. The discovery in the late 1990s of regulatory functions of short RNAs, such as miRNA (micro RNA) and siRNA (short interfering RNA), which play a key role in transcriptional and post-transcriptional gene silencing, have revolutionized genomic studies and drawn the research interest to other classes of ncRNAs [3]. Later studies in mammalian and human cells revealed the functions of long noncoding RNAs in epigenetic regulation of gene expression [4]. lncRNAs are classified as transcripts longer than 200 nt with no protein-coding potential or with low protein-coding potential. LncRNAs can exert their effects in *cis* by regulating the expression of neighboring genes and in *trans* by affecting multiple genes across the whole genome. At the molecular level, lncRNAs can interact with DNA, RNA, and proteins. A detailed mechanism of lncRNA molecular function have been reviewed in [1,5]. For a long time, little progress was made in the research studies of lncRNA function in plants and this progress accelerated in the recent decade with the wide application of next generation sequencing (NGS) into genomic studies. Although the NGS allowed for identification of thousands of lncRNA transcripts in different plant species, only a very small fraction of them was experimentally validated. Biological functions of most of the plants’ lncRNAs have been discovered in the model species *Arabidopsis thaliana* (L.) Heynh. LncRNAs are involved in different developmental processes, such as flowering, vernalization, root development, pollen development, seedling morphogenesis, phosphate homeostasis, as well as in the response to different biotic and abiotic stress conditions (reviewed in [6,7,8]). Because of the crucial role of lncRNAs in plant development and the stress response, they have a high application potential in crop breeding programs. However, this application must be preceded by extensive functional studies, as only a few lncRNAs have been functionally validated in important cereal crops. This research can now be greatly accelerated with the use of genome editing techniques, such as CRISPR/Cas9. In recent years genome editing has become a widely used method in genomic studies in plants, as efficient CRISPR/Cas9 protocols have been developed even for wheat and barley [9,10]. Barley is the fourth most important cereal crop after wheat, rice, and maize, which is cultivated worldwide. Although the number of identified barley lncRNAs in the PLncDB database [11] is 25,884 (2021), there are only a few reports concerning lncRNAs research and they are mostly focused on the stress response. For example, Karlik and Gozukirmizi [12] identified up-regulation of the sense lncRNA *AK370814* under salinity stress. Qiu et al. [13] identified 535 lncRNAs responsive to drought conditions in the Tibetan wild barley cultivar. lncRNA regulation in barley has also been evaluated in nutrient excess or deficiency conditions. For instance, Unver and Tombuloglu [14] analyzed the expression pattern of novel lncRNAs under excessive boron treatment, and Chen et al. [15] identified 487 low nitrogen stress responsive lncRNAs in a barley landrace. Recently, Barakte et al. [16] revealed the potential role of lncRNAs at the meiotic phase in anther. Although the role of lncRNAs in the stress response is very important, there is a lack of genome-wide studies of lncRNA regulation during barley development. A relatively low and highly tissue-specific expression pattern of lncRNAs, and their low evolutionary conservation, are major constraints in identification and functional analysis of lncRNAs in plants. To a large extent, these constraints can be overcome with the still improving DNA and RNA sequencing technology. Thanks to this improvement, high quality genome references are available for more crop plants, even for species with large and complex genomes, such as wheat [17]. Moreover, as a result of wide application of RNAseq technology in recent years, the number of publicly available transcriptomic data increased rapidly [18]. These large datasets of transcript sequences obtained from different plant tissues and at different experimental conditions allow for analysis of lncRNAs in the whole plant [7,11,19]. In this study, we performed global identification and evaluation of barley lncRNAs based on RNAseq data obtained from a range of barley tissues at different developmental stages. In particular, we focused on the expression patterns of lncRNAs and identification of their potential target genes during two important developmental stages, which have a direct impact on grain yield. The first analyzed stage is the shoot apex development at the transition from vegetative to generative phase. This phase is critical for the proper development of floral organs and is sensitive inter alia to temperature and photoperiod. At the beginning of the generative phase, there is strong competition for assimilates between developing tillers and main stem spikelets. This competition determines the survival of tillers and the number of fertile spikelets per spike, and consequently the final number of grains per plant [20]. The second analyzed stage is the grain development between 3 and 18 DPA (days post-anthesis). In this period, the balance between cell proliferation and grain filling rate determines the final grain weight [21]. The understanding of the role of lncRNAs in these developmental processes can be used in the future for improvement of barley yield. We also analyzed the tissue specificity of lncRNA expression and determined their evolutionary conservation between *Arabidopsis* and more closely related monocot species.

## 2. Results

### 2.1. Identification and Basic Characterization of Barley LncRNAs

For global identification of lncRNAs in barley we used 53 RNAseq libraries available in the SRA (Sequence Read Archive) database, representing nine different tissues and organs, both vegetative and generative, at different developmental stages. These libraries contained from two to four biological replicates of each tissue or developmental stage, which accounted for the total number of 1,474,501,022 sequence reads that were used for the analysis. The detailed description of each RNAseq library is in Supplementary Table S1.

**Figure 1:**
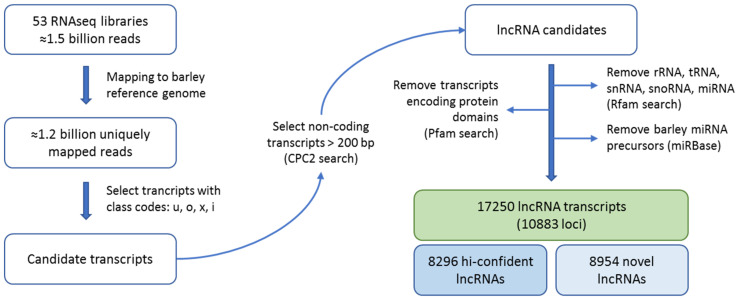
Schematic representation of the procedure used for identification of barley lncRNAs.

The steps of the identification pipeline are presented in Figure 1. Each RNAseq library was individually mapped on the barley reference genome and the transcripts were assembled using StringTie, based on uniquely mapped reads. Next, the assembled transcripts were combined by Cuffmerge and compared to the reference annotation to assign transcripts’ class codes. For prediction of lncRNAs only transcript with the class code “u” (unknown, intergenic), “i” (full intron overlap), “o” (exon overlap) and “x” (overlap with the reference transcript in antisense orientation) and longer than 200 bp were selected, which gave a total of 26,274 initial lncRNA candidates.

**Figure 2:**
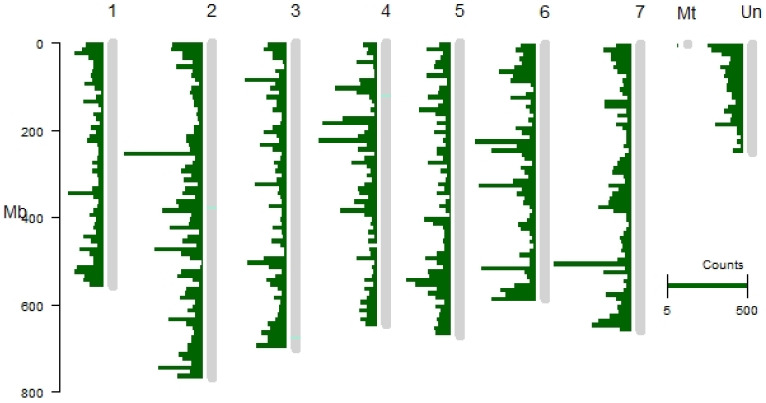
Distribution of lncRNA isoforms on barley chromosomes. Length of horizontal bars corresponds to the mapped read counts.

The next steps of the procedure were performed to filter out other noncoding RNA classes, such as rRNA, tRNA, snRNA, snoRNA, miRNA precursors, and potential protein-coding transcripts. In the latter case, we paid particular care to retain lncRNAs which overlap the protein-coding sequences in a sense orientation and distinguish them from mRNA transcripts. Candidate transcripts which indicated 90% sequence similarity and 80% alignment coverage with the protein-coding sequence were removed. For the remaining 1772 candidate transcripts that partially overlap the protein-coding sequences, we predicted all their possible reading frames using the getorf tool [22]. The resulting 3431 sequences were used as a query in the Pfam database to filter out candidate sequences containing protein domains. In summary, 17,250 transcript isoforms from 10,883 loci were identified as lncRNAs in the tested samples of barley (Supplementary File S1). Most identified transcripts, i.e., 15,543 (9788 loci), were classified as intergenic (class code “u”). From the remaining lncRNAs, approximately equal number was classified as exonic (816 transcripts, 556 loci, class code “o”) and NATs (natural antisense transcripts) (870 transcripts, 518 loci, class code “x”). The smallest group of lncRNAs, 21 transcripts (21 loci), were intronic overlaps (class code “i”). The identified lncRNAs are evenly distributed on all chromosomes, except the plastid genome, where none of the lncRNAs was mapped (Figure 2).

**Figure 3:**
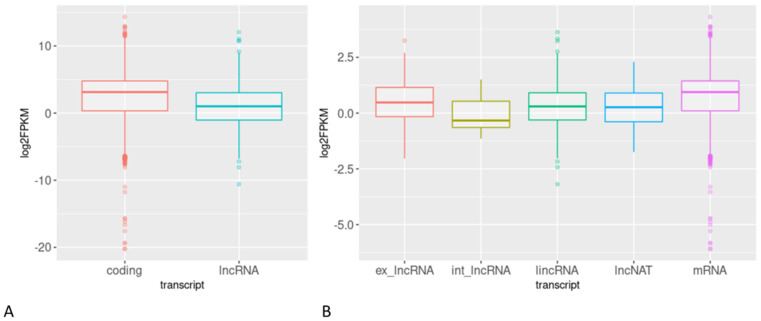
Expression levels of lncRNA and protein-coding gene transcripts. (**A**) Comparison of the FPKM values of all lncRNAs and coding transcripts; (**B**) comparison of the FPKM levels of exonic (ex-lncRNA), intronic (int_lncRNA), long intergenic (lincRNA) and antisense (lncNAT) lncRNA transcripts with mRNA transcripts.

Most of the lncRNAs indicated a low expression level in all analyzed tissues. As shown in Figure 3A, the mean FPKM (fragment per kilobase of exon per million fragments mapped) value of lncRNAs was several-fold lower than in coding genes. A similar comparison of the mean FPKM level of particular types of lncRNA transcripts showed that intronic lncRNAs had the lowest expression level, while the expression of exonic lncRNAs, long intergenic ncRNAs (lincRNAs) and NATs was at a similar level. mRNAs were the most abundant type of transcripts in all analyzed tissues and conditions (Figure 3B).

### 2.2. Evolutionary Conservation of Identified LncRNAs

To determine the evolutionary conservation of identified barley lncRNAs across plant species we performed blast analysis with the known lncRNAs from *Arabidopsis* and more closely related monocot species, such as maize, rice, and wheat. lncRNA sequences were acquired from the PLncDB database [11], which aggregates plant lncRNA sequences from RNAcentral and other databases. As expected, the biggest number of unique hits, 861, was found in wheat, which is the closest relative crop of barley. Only 7 and 3 hits were found in rice and maize, respectively, and no significant hit was found among *Arabidopsis* lncRNAs (Table 1 and Supplementary File S2).

We also checked which of the lncRNAs identified in this study have already been discovered in barley and we found 7920 unique hits with *H. vulgare* lncRNAs deposited in the PLncDB database, of which 5829 showed 100% sequence identity (Supplementary File S2). After combining transcripts homologous to rice, maize, wheat, and barley lncRNAs we excluded those that were repeated. As a result, 8296 lncRNA transcripts were selected, which we consider as hi-confident barley lncRNAs, while the remaining 8954, which do not show homology with the known plants lncRNAs, are considered to be novel barley lncRNAs (Supplementary File S2). The sequences of novel lncRNAs can be accessed in PLncDB website using JBrowse tool; the set is annotated as Long-Noncoding Gasparis et al. (http://plncdb.tobaccodb.org/JBrowse, accessed on 21 April 2021).

### 2.3. Tissue Specificity of Barley LncRNAs

**Figure 4:**
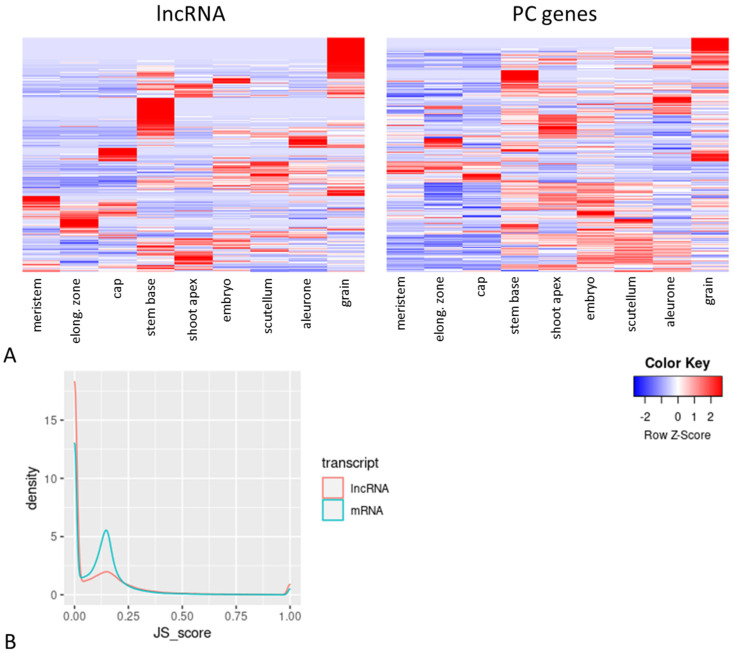
Evaluation of tissue specificity of lncRNA expression. (**A**) Expression patterns of lncRNAs and PC genes in nine different barley tissues; higher z–score (red colors) indicates higher tissue specificity; z–scores were calculated based on the mean FPKM values of each tissue. (**B**) Density plot of the JS–score calculated for PC genes and lncRNAs; JS–score 0 means no tissue specificity and 1 means unique expression in only one tissue.

Tissue specificity of expression of lncRNAs was first evaluated in all nine tissue samples (Supplementary File S3). As shown in Figure 4A, the expression pattern of lncRNAs is more differentiated than that of protein-coding (PC) genes and consists of a few main clusters that are specifically expressed in particular tissues (red color). We also calculated the Jensen-Shannon (JS) score based on the normalized FPKM values, which is used for estimation of tissue specificity of expression. The majority of lncRNAs had a slightly higher JS-score than PC genes; however, the number of lncRNAs with the score 1, which means 100% specificity, was higher than the number of PC genes (Figure 4B).

**Figure 5:**
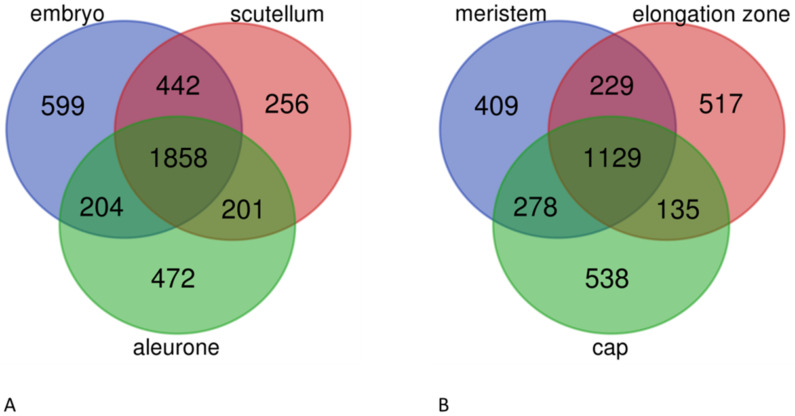
Number of co-expressed and uniquely expressed lncRNA loci in different parts of grain (**A**) and root (**B**).

The expression of lncRNAs was further evaluated in tissue samples derived from the same experiment. We selected lncRNA loci with the FPKM value above 0.5 and compared their expression between embryo, scutellum, and aleurone in grains and between meristem, elongation zone, and cap in roots. As shown in Figure 5A, 1858 lncRNA loci are expressed in all grain tissues, while 1327 loci are expressed exclusively in only one of three tissues, including 599 loci in embryo, 256 loci in scutellum and 472 loci in aleurone (Supplementary File S4). In the case of roots, the expression of lncRNA loci also indicates a strong tissue specific pattern. The number of lncRNAs that are expressed exclusively in either meristem (409) or elongation zone (517), or cap (538), is greater than the number of lncRNAs that do not show tissue specificity of expression (Figure 3B and Supplementary File S4).

### 2.4. Developmental Regulation of LncRNAs

**Figure 6:**
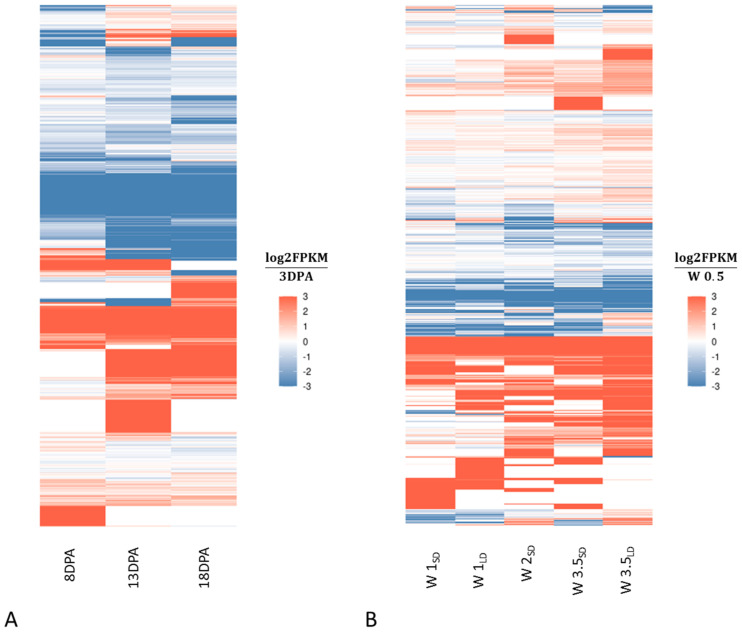
Changes in expression of lncRNAs in the developing grains in relation to 3 DPA stage (**A**) and in the developing shoot apices in relation to stage W 0.5 (**B**); red color indicates up-regulation and blue color indicates down-regulation of lncRNA expression; SD, short day conditions; LD, log day conditions. Heatmaps were drawn based on normalized log_2_FPKM values from all sample replicates.

Developmental regulation of identified lncRNAs was examined in the developing grains at 3, 8, 13 and 18 days post-anthesis (DPA) in cultivar Clipper, and in the developing shoot apex in cultivar Scarlett at W 0.5, W 1, W 2, W 3.5, where W 1 stage denotes the apex at the transition from the vegetative to the generative phase on the Waddington scale [23]. Significant differentially expressed lncRNAs (*p*-value < 0.05, *q*-value < 0.05) were determined by pairwise comparisons between consecutive developmental stages. In the developing grains, the number of differentially expressed lncRNA loci ranged between 752 (13 DPA vs. 18 DPA) and 1095 (8 DPA vs. 13 DPA) (Supplementary File S5). The heatmap in Figure 6A illustrates time-course changes of differential expression in relation to initial 3 DPA stage. The number of up-regulated lncRNAs increased rapidly at 13 and 18 DPA (red bars on the heatmap), while the differences between down-regulated lncRNAs were less evident.

In the developing shoot apex, the number of differentially expressed lncRNAs increased during the development from 94 at W 0.5 up to 619 at the final stages (W 3.5) (Supplementary File S5). Figure 6B shows the time-course changes of differential expression in relation to initial W 0.5 stage. The number of up-regulated lncRNAs increased gradually between W 0.5 and W 3.5. The pattern of up-regulated lncRNAs is the most differentiated at W 0.2 and W 3.5. There are also differences between samples of the same stage but exposed to short day or long day lighting conditions. As with grain development, the differences between down-regulated lncRNAs are less evident.

### 2.5. Cis-Regulation of LncRNA Neighboring Genes

**Figure 7:**
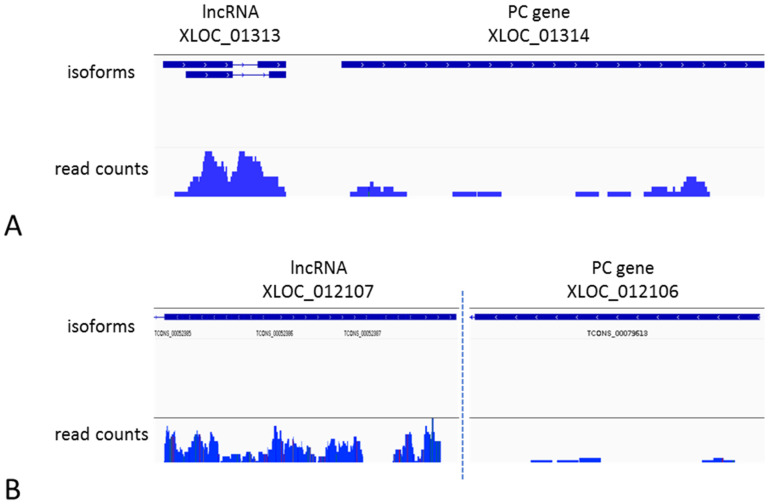
Examples of *cis*-down-regulation of PC genes by the neighboring lncRNA loci in developing grains at 8 DPA (**A**) and shoot apex at W 3.5 (**B**); length of vertical bars corresponds to the mapped read counts.

Regulatory function of barley lncRNAs was analyzed in the developing grains and shoot apex. To identify the regulation in *cis* manner, we first selected lncRNAs located up to 10 kb upstream or downstream of protein-coding genes. Using bedtools, we found 4463 unique lncRNA loci overlapping with 5605 loci of protein-coding genes. As some of the lncRNAs overlapped with more than one PC gene locus within the 10 kb window, the final number of lncRNA-PC gene pairs was 6882. Next, we estimated the correlation of expression within lncRNA-PC gene pairs and selected significantly correlated pairs using the cutoff of *p*-value < 0.05 and *r* < −0.8 for negative correlation and *r* > 0.8 for positive correlation. In the developing grains 78 negatively and 346 positively correlated PC genes were identified (Supplementary File S6). In the developing shoot apex, the number of negatively regulated PC genes was 60 and positive regulation was found for 116 PC genes (Supplementary Files S7). Figure 7 shows examples of negative correlation, in which increased expression of lncRNA was associated with decreased expression of PC genes. We also performed functional enrichment analysis of PC genes regulated by lncRNAs using the Mercator tool [24]. This tool assigns the biological function to proteins based on MapMan ontology, which was designed specifically for plants. In the developing grains, 464 protein-coding transcripts were assigned to 22 functional categories (Supplementary Figure S1 and File S6). Most of the classified transcripts (104) are involved in RNA biosynthesis (Bin code 15), in multi-process regulation of the lipid regulatory system (51 transcripts, Bin code 25.7) or show enzymatic functions (47 transcripts, Bin code 50). A significantly higher number of PC transcripts was characterized in the case of shoot apex (Supplementary File S6). 1765 PC transcripts were assigned to 22 different categories of biological function (Supplementary Figure S1). As with grains, the highest number of transcripts (329) are associated with RNA biosynthesis (Supplementary Figure S1). The second largest group is solute transport (Bincode 24, 227 transcripts). A relatively high number of transcripts was assigned to protein modification (Bin code 18) and protein homeostasis (Bin code 19) groups (198 and 168 assigned transcripts, respectively). A full description of biological functions and the number of transcript assigned to each Bin category are provided in the Supplementary File S7.

### 2.6. Trans-Regulation of PC Genes by LncRNAs

lncRNAs may also indicate *trans*-acting regulation of protein-coding genes. To identify barley lncRNAs that may be involved in *trans*-regulation of PC genes, we analyzed the correlation of expression between lncRNAs and PC genes in the developing grains and shoot apex. Because of the very large number of PC genes and to avoid false positive correlations, we selected only PC genes that showed the highest variance of expression, i.e., their expression changed between consecutive developmental stages. Consequently, approximately 5000 PC genes were selected for comparison with the approximately equal number of lncRNAs, which were expressed in at least two developmental stages at the 0.05 FPKM level or above. Significantly correlated pairs were selected for *r* ≥ 0.95 and *r* ≤ −0.95 for positive and negative correlation respectively, at a *p*-value < 0.05. As a result, 425 positively and 115 negatively correlated lncRNA loci were found in developing grains (Supplementary File S8). Since all the lncRNAs were correlated with multiple PC genes, the total number of significantly correlated lncRNA-PC gene pairs was 9449. The total number of lncRNA-PC gene pairs was lower in the developing shoot apex (7891), although the number of unique positively and negatively correlated lncRNAs was higher than in grains (940 and 796 respectively) (Supplementary File S9). Once again, we performed the functional enrichment analysis for PC genes regulated by lncRNAs using the Mercator tool. As shown in Supplementary Figures S3 and S4, positively and negatively regulated PC genes from both tissues were assigned to all 28 biological function categories and were more evenly distributed in those categories than *cis*-regulated genes.

## 3. Discussion

Advances in RNA sequencing technologies have greatly contributed to the knowledge of complex biological processes in plants. Studies at the genome-wide and whole transcriptome level allow one to determine the interactions between genes and transcription factors and to identify biological function of different classes of noncoding RNAs. However, for many reasons, the RNAseq-based identification of lncRNAs is more challenging than in the case of protein-coding genes. First, the majority of lncRNAs are in intergenic, unannotated genomic regions; thus, in a typical RNAseq pipeline, such transcripts are either undetected or classified as novel transcripts and excluded from the downstream analyses. Secondly, because of the still relatively high cost of NGS and the relatively difficult bioinformatics analysis requiring powerful computers, in many laboratories RNAseq analysis is outsourced as a commercial service. Usually, such services include basic in silico analysis of RNAseq data; however, it is focused on protein-coding genes and does not include lncRNA identification, so a large part of valuable data is lost. Finally, because of very low expression of lncRNAs, their identification and analysis is particularly challenging in crop plants with large genomes, such as wheat, oat, barley, or maize. It complicates both experimental studies, such as RT-qPCR validation of expression, and in silico analyses, where it is difficult to spot the subtle changes of lncRNA expression in the enormously large datasets. In this study, we followed procedures of lncRNA identification and characterization that have been successfully used in plants [25,26,27,28], and when necessary, we introduce some modifications. We used combination of different bioinformatic tools, rather than single software packages, such as TopHat or Cufflinks, which are optimized for human sized or smaller genomes. For example, we used STAR (Spliced Transcripts Alignment to a Reference) software [29] for mapping of the RNAseq reads, which is fast and can handle large genomes using less RAM memory, and StringTie, which is known for improved precision in transcript assembly [30]. We believe that such a combination is better suited to the specific features of the large barley genome (~5.3 Gb of haploid genome as compared to 3.1 Gb of human genome and 135 Mb of *Arabidopsis* genome), where some of its parts remain still unannotated.

As with other plants’ lncRNAs, the barley lncRNAs identified in this study are equally distributed across the whole genome, and indicate low expression, weak evolutionary conservation, and tissue specificity of expression. Although the range of expression level of some barley lncRNAs is wide, depending on tissue and/or developmental stage, the expression level of the majority of lncRNAs is several-fold lower than expression of protein-coding genes. This agrees with the previous observations in plants; e.g., in *Arabidopsis* the transcript levels of lincRNAs were 30–60 fold lower than mRNA transcripts [5]. Another typical property of ncRNA is its weak evolutionary conservation. Homologous lncRNA sequences can be found only between closely related species, although this homology rapidly decreases with the genetic distance. Studies in mammals also showed that lncRNAs are rarely orthologous and equivalent in position of loci [31]. It should be noted, however, that despite weak sequence similarity, lncRNAs shows functional conservation. Barley lncRNAs showed the highest sequence homology with wheat lncRNAs, which is not surprising as both species are closely related members of the Triticeae tribe. However, even within the Poaceae family, the evolutionary conservation of lncRNAs is significantly weaker between more distant relatives, as we found only 7 and 3 homologous sequences in rice and maize, respectively. In terms of evolutionary conservation, lncRNAs are similar to miRNA precursor sequences, which also are weakly conserved, in contrast to mature miRNAs [32]. In sum, we identified 879 barley lncRNAs that are homologous to wheat, rice, and maize lncRNAs. This information may be useful in future studies when more lncRNAs are experimentally validated. The function of homologous lncRNAs in other species could be then predicted based on sequence homology with the validated lncRNA. Moreover, 7920 of lncRNA transcripts analyzed in this study have been previously discovered in barley and deposited in lncRNA databases. These lncRNAs together with 879 homologous lncRNAs can be considered to be high-confident lncRNAs, since they have been previously identified by other researchers and were confirmed in this study.

### 3.1. Expression of Barley LncRNAs Is Tissue Specific

Global studies in different plant species demonstrated that lncRNAs indicate high tissue specificity of expression [33,34,35]. Here, we decided to analyze the expression of lncRNAs in different parts of grains and roots, which very often in transcriptomic studies are treated as a whole single tissue. Our data show that even in the same organ some lncRNAs may be specifically expressed in a certain part, while the same lncRNAs remain inactive in the neighboring cells. Specific expression of some lncRNAs in the root meristem, elongation zone, and cap may indicate that these lncRNAs are involved in cell differentiation and proper root formation. It is especially intriguing that even in the embryo, despite close physical proximity between cells, there are nearly 600 lncRNAs that are expressed only in embryo axes and 256 that are expressed only in scutellum. As with root meristem, these 600 lncRNAs from germinating embryo axes may be involved in cell differentiation and organ formation. The tissue specificity of lncRNA expression may have serious implications in transcriptomic studies and in the way of how tissue samples should be collected and RNAseq libraries prepared. In some cases, the results of RNAseq analysis of lncRNAs in the developing grains may be more robust and biologically relevant if the RNA libraries are prepared from separate tissues of the grain than from the whole grain. Analogically, the response of lncRNAs to drought stress conditions may be completely different in the different parts of the roots.

### 3.2. Stage of Development-Related lncRNAs

Many lncRNAs are involved in regulation of plant development. The most known and well characterized examples are the COOLAIR in *Arabidopsis* and COLDAIR in *B. distachyon*, which regulate flowering time by histone modification (reviewed in [6]). We analyzed the changes of lncRNA expression during the development of grains and shoot in the RNAseq libraries which were derived from the same cultivars and experimental conditions. In both cases we observed several hundred lncRNAs that were differentially expressed between the successive stages. In the developing grains, the expression of some lncRNAs increases significantly between 8 and 13 DPA. This is a critical stage of grain development when the transition from the cell proliferation phase to the starch accumulation phase occurs and its course has an influence on the final grain size and weight [19]. This differential expression of lncRNAs coincides with the whole transcriptome dynamics that were observed previously in the RNAseq data derived from the same samples by Bian et al. [36]. Elucidation of the role of lncRNAs during this process may have an essential application value for improvement of grain yield. We also observed up-regulation of some lncRNAs in the developing shoot apex during transition from the vegetative to the generative phase. This is also a critical phase influencing inflorescence development and flowering time, and consequently strongly affecting yield in barley. Here we analyzed a few RNAseq libraries from the study of Digel et al. [37], in which the effects of photoperiod on barley development were analyzed at the transcriptome level. The authors found that differences in the photoperiod response were associated with the induction of FLOWERING LOCUS T orthologs in barley, FT1 in leaves and FT2 in shoot apices. The proper inflorescence development was correlated with the up-regulation of FT2 locus and transcripts related to floral organ development, phytohormones, and cell cycle regulation. We suppose that lncRNAs may also be involved in this regulatory network, as we can see differences in lncRNA expression not only between successive development stages of the shoot apex but also between samples exposed to different daylight conditions.

### 3.3. Cis-Regulation of PC Genes by LncRNAs

In gene regulation, lncRNAs can act in both a *cis* and a *trans*-manner. *Cis*-regulation is related to genes which are overlapped or are adjacent to lncRNA loci. We analyzed the *cis*-regulatory mechanism in the developing grains and shoots among the “genic” lncRNAs, i.e., lncRNAs overlapping with or adjacent to the PC genes within a 10 kb upstream or downstream window. Both positive and negative correlations were observed among lncRNA-PC gene pairs. The positive correlation may simply result from the co-transcription of the overlapping exonic or intronic lncRNAs with the PC gene. However, lncRNAs can also act as transcription activators by capturing transcription factors to the promoter region of the neighboring PC gene. Such enhancer RNAs (eRNAs) that act in *cis* have been observed in mammalian cells [38,39], and recently the *cis*-NAT_PHO1;2_ lncRNA, which enhances the expression of *PHO1;2* gene, has been identified in rice [40]. It is difficult to predict whether the positive correlation is a regulatory mechanism or simply a co-expression and each individual case would need to be experimentally validated. A more interesting case is the negative *cis*-regulation of PC genes, as this is a commonly observed mechanism in plants and animals. In this mechanism, lncRNA transcripts, in most cases natural antisense transcripts (NATs), are precursors of siRNA molecules in the RNA interference mechanism (RNAi). Consequently, the target PC gene is down-regulated in the siRNA pathway by mRNA degradation or translation inhibition [41]. A well-described example of a NAT transcript in barley is *HvCesA6*, which is a precursor for siRNAs that target the *CesA6* gene and regulate cell wall synthesis [42]. The functional enrichment analysis of negatively regulated PC genes, both in grains and shoots, indicated that most of them function in different processes of RNA biosynthesis. Another numerous group comprised enzyme encoding genes, which suggests that enzyme activity modulation by lncRNAs, similarly as in the *HvCesA6* example, is a more common mechanism in barley.

### 3.4. Trans-Regulation of PC Genes by LncRNAs

*Trans*-regulation of PC genes by lncRNAs is a more complex mechanism than *cis*-regulation, as the lncRNAs can act at multiple levels by interaction with DNA, RNA, and proteins. In the most known examples, lncRNAs can act as target decoys or sponges for siRNA, miRNA, or mRNA binding proteins, which prevents the mRNA from degradation or translational inhibition. lncRNAs also function as scaffolds for different chromatin modifying or chromatin remodeling proteins. Such complexes can modulate the expression of the target genes by chromatin modifications, e.g., methylation, or by remodeling the chromatin structure. In addition, finally, lncRNA transcripts may serve as precursors for siRNA in the RNA-directed DNA methylation (RdDM) pathway of DNA methylation (the above examples are reviewed in detail in [5]).

In silico prediction of *trans*-acting lncRNAs is particularly challenging, because theoretically every lncRNA can interact with multiple PC genes; thus, the number of possible interactions at the whole genome level is enormous. There are some computational tools designed to identify statistically significant interactions in such large co-expression networks, and one of the most commonly used is weighted gene correlation network analysis (WGCNA) [43]. Unfortunately, we could not use this tool in this study, as it requires at least 20 samples from the same experiment. Therefore, for correlation analysis we selected only lncRNA-PC gene pairs with the highest variance of expression between the successive developmental stages. Moreover, we applied a very stringent cutoff value for correlation, i.e., *r* > 0.95, while *r* > 0.8 is considered to be biologically relevant. Regardless of the statistical methods used, each in silico prediction requires experimental evidence, and for this reason we consider our results rather as a pre-selection of *trans*-acting lncRNAs, similarly as in other global studies of lncRNA [33]. Together with the functional classification of target PC genes (Supplementary File S7), such a preselected set could be useful in more detailed transcriptomic studies in barley.

## 4. Materials and Methods

### 4.1. Data Retrieval

RNAseq data were retrieved from NCBI (National Center for Biotechnology Information) Sequence Reads Archive [44]. Raw sequence reads from 53 SRA experiments were downloaded and converted to the fastq format using SRA Toolkit (https://trace.ncbi.nlm.nih.gov/Traces/sra/sra.cgi, accessed on 20 January 2021). RNA libraries were derived from the root meristem, root elongation zone, root cap, stem base, shoot apex, aleurone, scutellum, embryo axe, and from the whole grains. RNA libraries derived from root tissues were prepared using random primers and the remaining libraries were prepared using oligo-dT. The accession numbers and basic description of each SRA experiment are shown in Supplementary Table S1.

IBSC_v2 [45] assembly of the barley reference genome was used in this study. DNA sequence of the genome in fasta format and gene annotation in gtf format were downloaded from Ensembl Plants [46].

### 4.2. Transcriptome Assembly and Expression Analysis

The quality of raw sequence reads was first inspected using the FastQC tool [47] (https://www.bioinformatics.babraham.ac.uk/projects/fastqc/, accessed on 20 January 2021). If required, the sequences were processed by the Trimmomatic tool [48] to remove Illumina adaptor sequences and low-quality reads (Q < 30). STAR software [29] was used for genome index creation and for mapping of each individual RNA library to the reference genome. Transcripts were assembled from the aligned reads using StirngTie [30]. Next, the assembled transcripts from each RNA library were merged to one file using the Cuffmerge tool from the Cufflinks 2.0 package [49], and then compared to the reference genome to assign class codes to each transcript using the Cuffcompare tool from the same package. The Cuffdiff tool from the Cufflinks 2.0 package was used to capture the differentially expressed genes from the pairwise comparisons of the samples. The output of the Cuffdiff tool was exported to the CummeRbund R package to read the expression level and differentially expressed genes. The gene expression level was calculated as fragment per kilobase of exon per million fragments mapped (FPKM) and the differential gene expression was calculated as log_2_ fold change of the FPKM value between compared samples. The function csHeatmap() from the CummeRbund R package was used to draw heatmap plots.

### 4.3. Identification of lncRNA Transcripts

To identify lncRNA transcripts in the analyzed transcriptome, we used a similar strategy to those described previously [25,26,27] with some modifications. First, only the transcripts with the class codes “u” (unknown, intergenic), “i” (full intron overlap), “o” (exon overlap) and “x” (overlap with the reference transcript in antisense orientation, NAT transcript) were selected for the analysis and the fasta file containing the selected transcript sequences was created using the gffread tool from the Cufflinks 2.0 package. The sequences were then submitted to the online coding potential calculator tool, CPC2 [50] (http://cpc2.gao-lab.org/, accessed on 20 January 2021), to filter out potentially coding sequences. The remaining sequences longer than 200 bp were used as a query in the Rfam database (https://rfam.xfam.org/, accessed on 30 March 2021) to filter out other functional RNAs, such as rRNA (ribosomal RNA), tRNA (transport RNA), snRNA (small nuclear RNA), snoRNA (small nucleolar RNA) and miRNA precursors. Additionally, barley specific rRNA, tRNA, snRNA, snoRNA and MIR precursors were downloaded from Ensembl Plants and used as a query to find and remove additional, functional RNAs within the lncRNA candidates. The blastn search was performed using the standalone NCBI blastn-2.9.0-2 tool with the E-value < 0.005. A similar search was performed for 69 miRNA precursors of barley downloaded from the miRBase database (http://mirbase.org, accessed on 30 March 2021) [51]. Next, the candidate lncRNA sequences were submitted to a Pfam [52] search to identify transcripts that encode any conserved protein domains with the E-value < 0.005. To retain sense lncRNAs that overlap with the protein-coding exons, we selected all sense candidate lncRNAs and predicted their open reading frames (ORF) in sense orientation, using the getorf tool from the EMBOSS 5.0 package [22]. Sequences translated from these ORFs were submitted once again to Pfam search to filter out sequences encoding protein domains. Distribution of lncRNAs on the barley genome was visualized using the chromPlot R package [53]

### 4.4. Evaluation of lncRNA Tissue Specificity

Z-scores and Jensen-Shannon scores (JS) were calculated based on the mean FPKM values from each tissue using the tspex Python script (https://github.com/apcamargo/tspex/, accessed on 30 March 2021) [54]. Heatmap plots and a density plot were drawn using the ggplot2 R package.

### 4.5. Analysis of Evolutionary Conservation of LncRNAs

To determine the conservation of the identified lncRNAs, they were compared with the lncRNA sequences from *A. thaliana*, *O. sativa*, *Z. mays* and *T. aestivum* downloaded from the PLncDB database (http://plncdb.tobaccodb.org/, accessed on 20 January 2021) [11]. The blastn search was performed using the standalone blastn-2.9.0-2 tool with E-value < 0.005. Sequences indicating above 80% identity with the annotated lncRNAs were considered to be conserved. We also blasted the identified lncRNAs with the barley lncRNAs from PLncDB using the parameters above. lncRNAs of which the sequence identity with the annotated lncRNAs was below 95% and the alignment did not cover the whole sequence length were considered to be novel barley lncRNAs.

### 4.6. Identification of Cis- and Trans-Acting LncRNAs

To identify regulation of PC genes by lncRNAs in *cis*, we first selected lncRNAs and the neighboring PC genes at the distance of 10 kb using Bedtools [55]. Next, the correlation of mean FPKM values in lncRNA-PC gene pairs was estimated. The Pearson correlation coefficient was calculated using the cor() function in R. The genomic location of the neighboring loci was visualized using the IGV (Integrative Genomics Viewer) tool [56,57]. Similarly, the correlation of mean FPKM values was estimated between lncRNAs and other PC genes regulated in *trans*. The *cis*-regulated PC genes were excluded from this analysis. Moreover, we used the var() function in R to select only PC genes with the highest expression variance between successive developmental stages. The Pearson correlation coefficient was calculated using the function corPvalueStudent() from the R package WGCNA [43]. Finally, we performed functional enrichment analysis for the *cis*- and *trans*-regulated PC genes using the online Mercator4 v2.0 tool (https://plabipd.de/portal/mercator-sequence-annotation, accessed on 31 March 2021) [24].

## 5. Conclusions

In this study, we identified 17,250 lncRNA transcripts from 10,883 loci and analyzed their expression patterns in different barley tissues. Our results showed that barley lncRNAs are highly tissue specific and regulate the development in both vegetative and generative phases. We also identified hundreds of potential *cis* and *trans*-acting lncRNAs and annotated the biological function of thousands of target protein-coding genes of which expression was correlated with those lncRNAs. We believe that these results are a solid background for further studies of lncRNA functions in barley but also in wheat, as many lncRNAs from both species are homologous. The knowledge about biological function of lncRNAs may open new possibilities in the breeding efforts that are focused on yield improvement, which is still insufficient in the case of barley.

## Figures and Tables

**Table 1:** Identification of homologous lncRNAs in barley and selected plant species.

Species	No. Unique Hits in PLncDB	Total lncRNAs in PLncDB
*Arabidopsis thaliana*	0	13,599
Rice	7	11,565
Maize	3	32,397
Wheat	861	43,659
Barley	7920	25,884

## Data Availability

All data supporting the reported results are included within the article or its Supplementary Materials files except of novel lncRNA transcript sequences which are available in the PLncDB website using JBrowse tool (http://plncdb.tobaccodb.org/JBrowse, accessed on 21 April 2021).

## Supplementary Materials

The following are available online at https://www.mdpi.com/article/10.3390/ijms22105087/s1, Table S1: Description of the RNAseq libraries used in this study; Figure S1: Functional annotation of down-regulated PC genes in the developing grains and shoot apices; Figure S2: Functional annotation of up-regulated and down-regulated PC genes in the developing grains; Figure S3: Functional annotation of up-regulated and down-regulated PC genes in the developing shoot apices; File S1: List and genomic coordinates of all identified lncRNAs; File S2: List of conserved and novel barley lncRNAs; File S3: Evaluation of tissue specificity of lncRNA expression; File S4: List of lncRNA loci expressed in grain and root tissues; File S5: List of differentially expressed lncRNA loci in grains and shoots; File S6: Summary of correlation analysis of cis-acting lncRNAs in developing grains; File S7: Summary of correlation analysis of cis-acting lncRNAs in the developing shoot apices; File S8: Summary of correlation analysis of *trans*-acting lncRNAs in developing grains; File S9: Summary of correlation analysis of *trans*-acting lncRNAs in the developing shoot apices.
