# Supplementary material for: Genome-Wide Identification of Barley Long Noncoding RNAs and Analysis of Their Regulatory Interactions during Shoot and Grain Development
Source: Int J Mol Sci. 2021 May 11;22(10):5087. doi: 10.3390/ijms22105087 (PMC8150791; doi:10.3390/ijms22105087)
Supplement: Supplementary file 1 [file ijms-22-05087-s001.zip › Fig. S1.pdf]

A

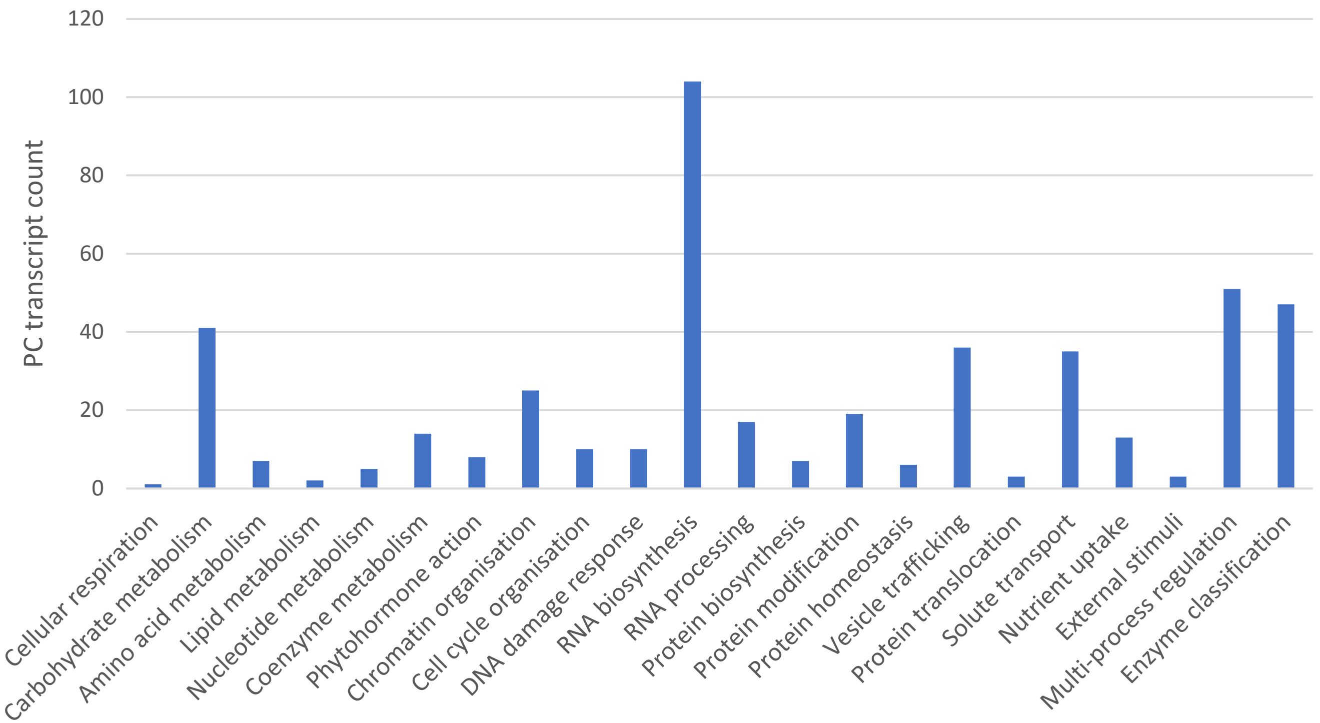

PC transcript count
120
100
80
60
40
20
0
Cellular respiration
Carbohydrate metabolism
Amino acid metabolism
Lipid metabolism
Nucleotide metabolism
Coenzyme metabolism
Phytohormone action
Chromatin organisation
Cell cycle organisation
DNA damage response
RNA biosynthesis
RNA processing
Protein biosynthesis
Protein modification
Protein homeostasis
Vesicle trafficking
Protein translocation
Solute transport
Nutrient uptake
External stimuli
Multi-process regulation
Enzyme classification

B

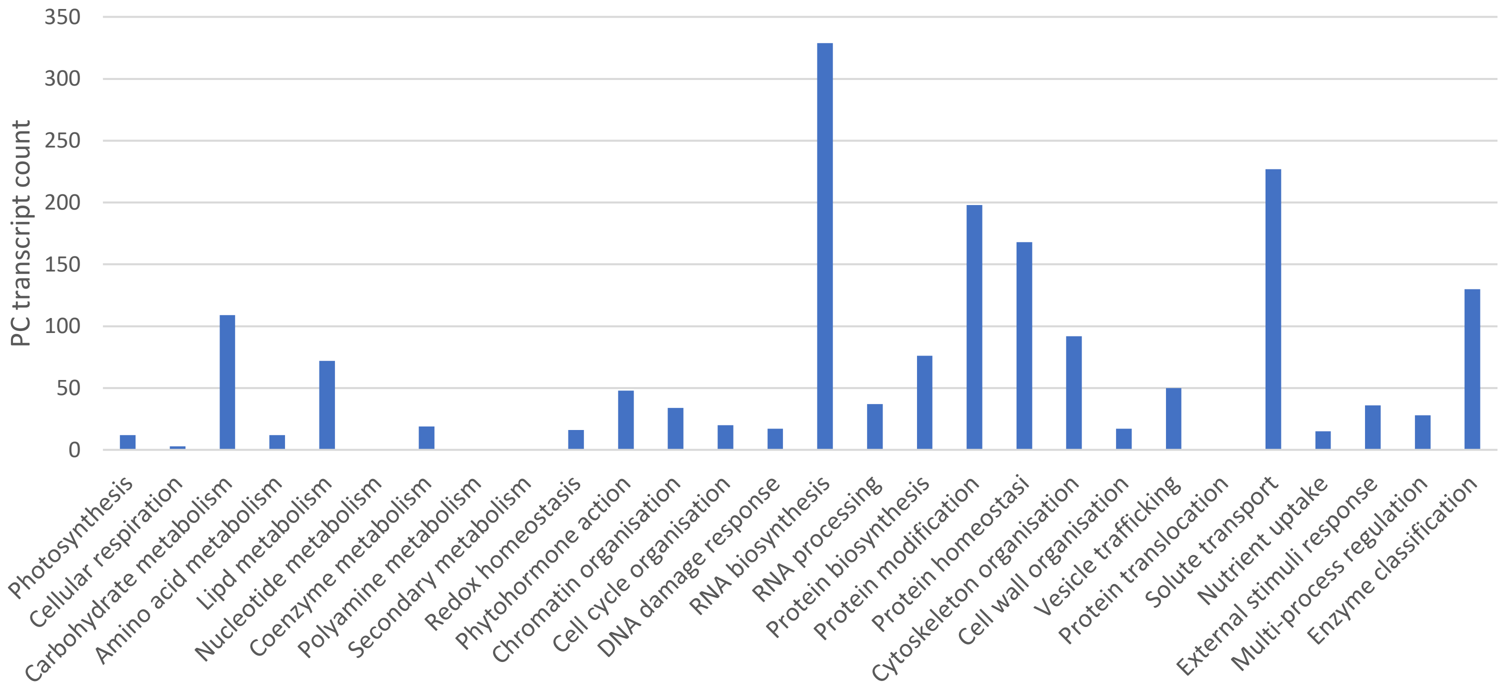

Fig. S1 Functional annotation of downregulated PC genes in the developing grains (A) and shoot apices (B).
