# Supplementary material for: Genome-Wide Identification of Barley Long Noncoding RNAs and Analysis of Their Regulatory Interactions during Shoot and Grain Development
Source: Int J Mol Sci. 2021 May 11;22(10):5087. doi: 10.3390/ijms22105087 (PMC8150791; doi:10.3390/ijms22105087)
Supplement: Supplementary file 1 [file ijms-22-05087-s001.zip › Fig. S2.pdf]

A

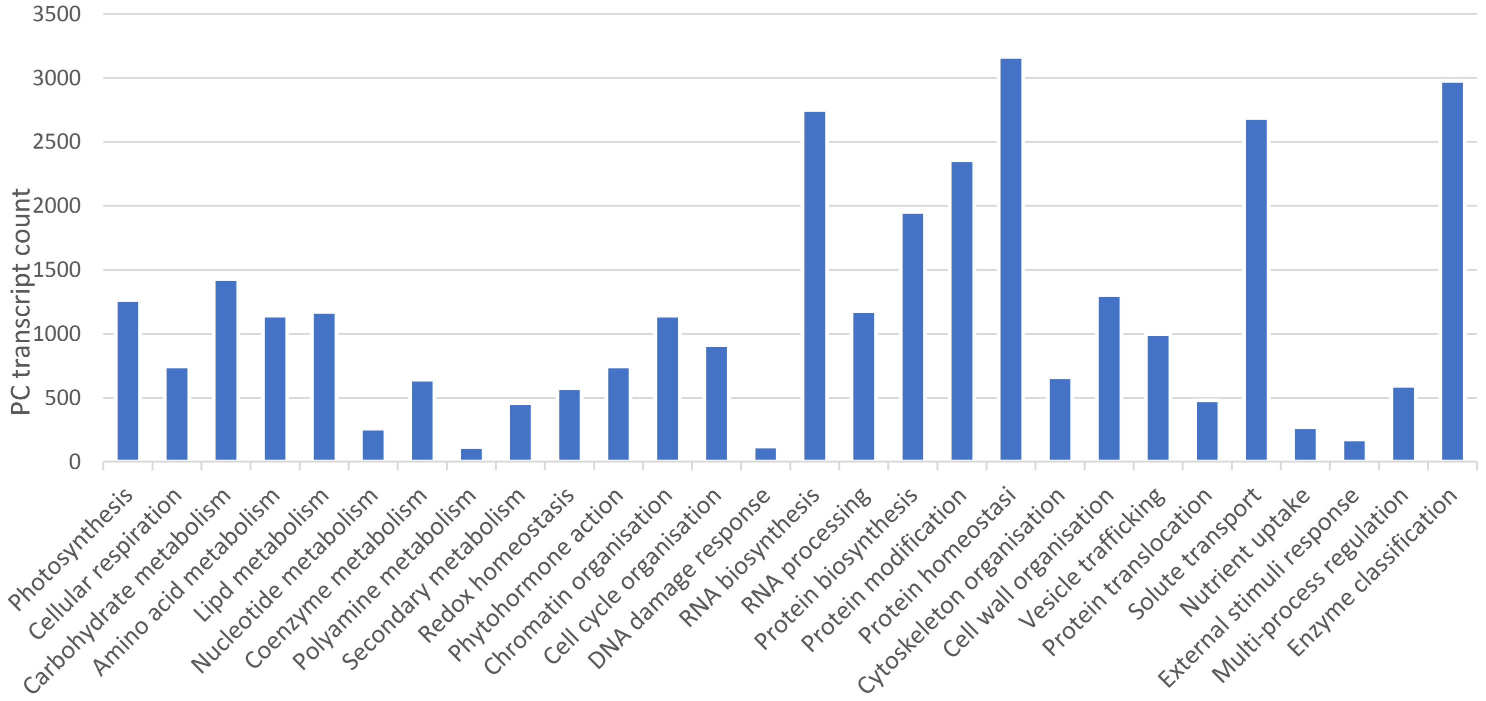

3500
3000
2500
2000
1500
1000
500
0
PC transcript count
Photosynthesis
Cellular respiration
Carbohydrate metabolism
Amino acid metabolism
Lipd metabolism
Nucleotide metabolism
Coenzyme metabolism
Polyamine metabolism
Secondary metabolism
Redox homeostasis
Phytohormone action
Chromatin organisation
Cell cycle organisation
DNA damage response
RNA biosynthesis
RNA processing
Protein biosynthesis
Protein modification
Protein homeostasi
Cytoskeleton organisation
Cell wall organisation
Vesicle trafficking
Protein translocation
Solute transport
Nutrient uptake
External stimuli response
Multi-process regulation
Enzyme classification

B

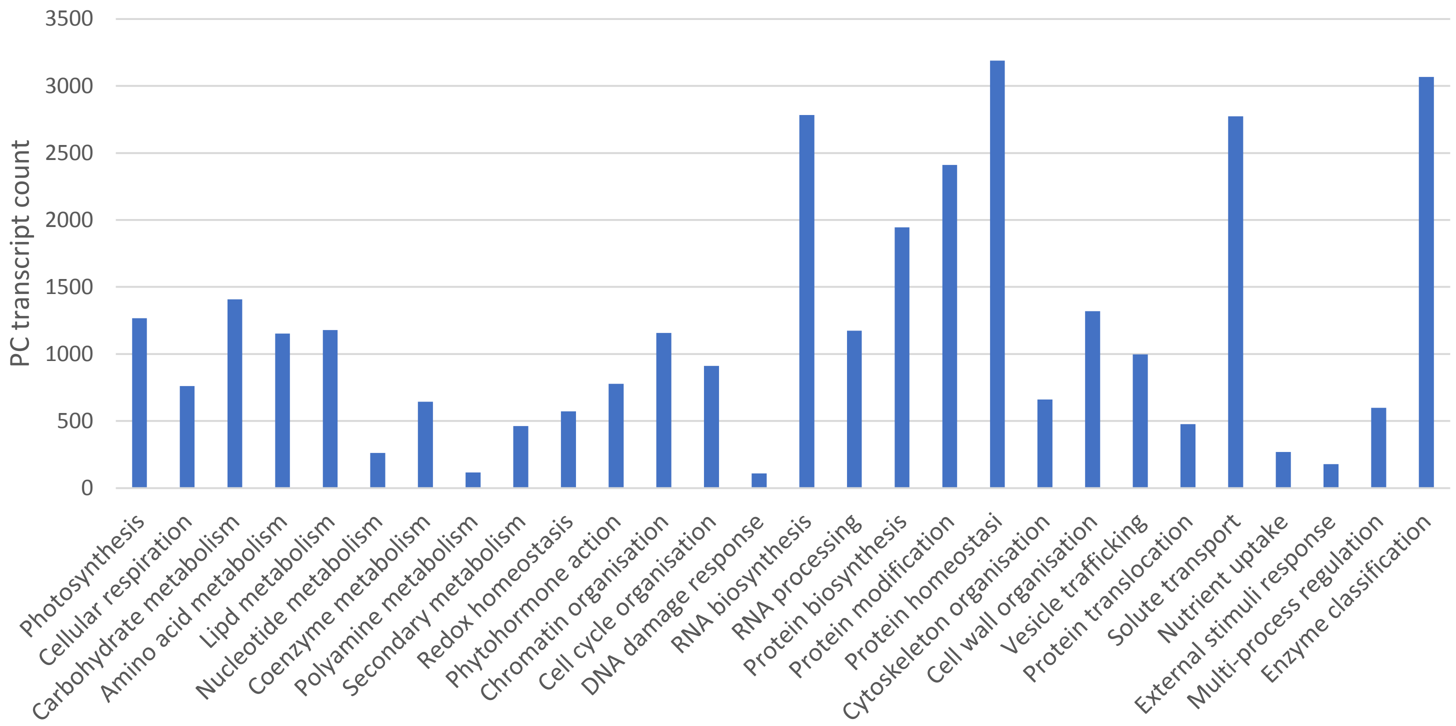

Fig. S2 Functional annotation of upregulated (A) and downregulated (B) PC genes in the developing grains.
