# Supplementary material for: Genome-Wide Identification of Barley Long Noncoding RNAs and Analysis of Their Regulatory Interactions during Shoot and Grain Development
Source: Int J Mol Sci. 2021 May 11;22(10):5087. doi: 10.3390/ijms22105087 (PMC8150791; doi:10.3390/ijms22105087)
Supplement: Supplementary file 1 [file ijms-22-05087-s001.zip › Table S1.pdf]

Table S1 Description of the RNAseq libraries used in this study

Run	Tissue	Dev. stage / replicate	RNA selection	Input reads	Uniquely mapped reads	Unique reads [%]
ERR781039	shoot apex	W 0.5_SD_rep1[a]	oligo-dT	15324805	13012586	84.91
ERR781040	shoot apex	W 0.5_SD_rep2	oligp-dT	5269320	4493415	85.28
ERR781041	shoot apex	W 0.5_SD_rep3	oligo-dT	3816694	3114026	81.59
ERR781045	shoot apex	W 1_LD_rep1[b]	oligo-dT	16201529	13746672	84.85
ERR781046	shoot apex	W 1_LD_rep2	oligo-dT	16233919	13396981	82.52
ERR781047	shoot apex	W 1_LD_rep3	oligo-dT	14865452	12570935	84.56
ERR781056	shoot apex	W 2_SD_rep1	oligo-dT	17361682	14981594	86.29
ERR781057	shoot apex	W 2_SD_rep2	oligo-dT	16887637	14570362	86.28
ERR781058	shoot apex	W 2_SD_rep3	oligo-dT	12466095	10717532	85.97
ERR781059	shoot apex	W 1_SD_rep1	oligo-dT	18945581	16220471	85.62
ERR781060	shoot apex	W 1_SD_rep2	oligo-dT	13076943	11197319	85.63
ERR781061	shoot apex	W 1_SD_rep3	oligo-dT	16407869	13875298	84.56
ERR781071	shoot apex	W 3.5_LD_rep1	oligo-dT	8012828	6676782	83.33
ERR781072	shoot apex	W 3.5_LD_rep2	oligo-dT	8282206	6948478	83.90
ERR781073	shoot apex	W 3.5_LD_rep3	oligo-dT	4756304	4035793	84.85
ERR781083	shoot apex	W 3.5_SD_rep1	oligo-dT	18186027	13953477	76.73
ERR781084	shoot apex	W 3.5_SD_rep2	oligo-dT	8326825	6900713	82.87
ERR781085	shoot apex	W 3.5_SD_rep3	oligo-dT	9119672	7494508	82.18
SRR10436257	root meristem	3 days_rep1	random primer	48823968	39742888	81.40
SRR10436260	root meristem	3 days_rep2	random primer	41437658	33374430	80.54
SRR10436263	root meristem	3 days_rep3	random primer	42945302	34687208	80.77
SRR10436266	root meristem	3 days_rep4	random primer	38800255	32212554	83.02
SRR10436255	root elong. zone	3 days_rep1	random primer	38895728	33613234	86.42
SRR10436259	root elong. zone	3 days_rep2	random primer	45637837	36645038	80.30
SRR10436262	root elong. zone	3 days_rep3	random primer	44297493	35518588	80.18
SRR10436265	root elong. zone	3 days_rep4	random primer	39482115	32928084	83.40
SRR10436261	root cap	3 days_rep1	random primer	43218267	35894054	83.05
SRR10436245	root cap	3 days_rep2	random primer	38087041	34247656	89.92
SRR10436264	root cap	3 days_rep3	random primer	41747316	33862556	81.11
SRR10436258	root cap	3 days_rep4	random primer	37518812	20841242	55.55
SRR10672924	scutellum	96h_rep1	oligo-dT	9108531	6343476	69.64
SRR10672925	scutellum	96h_rep2	oligo-dT	11023117	7536040	68.37
SRR10672926	scutellum	96h_rep3	oligo-dT	10388509	7405793	71.29
SRR10672941	embryo	96h_rep1	oligo-dT	10135243	6956122	68.63
SRR10672942	embryo	96h_rep2	oligo-dT	10593645	6657237	62.84
SRR10672943	embryo	96h_rep3	oligo-dT	11915785	8365528	70.21
SRR10672957	aleurone	96h_rep1	oligo-dT	12023832	8772699	72.96
SRR10672958	aleurone	96h_rep2	oligo-dT	11642221	8205159	70.48

Table S1 continued

Run	Tissue	Dev. stage / replicate	RNA selection	Input reads	Uniquely mapped reads	Unique reads [%]
SRR10672959	aleurone	96h_rep3	oligo-dT	8648885	6459005	74.68
SRR4380201	stem base	1DAG_rep1[c]	oligo-dT	76177362	60309534	79.17
SRR4380202	stem base	1DAG_rep2	oligo-dT	63227909	50203113	79.40
SRR4380203	stem base	1DAG_rep3	oligo-dT	60991125	48577407	79.65
SRR4380204	stem base	10DAG_rep1	oligo-dT	77896964	64391970	82.66
SRR4380205	stem base	10DAG_rep2	oligo-dT	54220130	44504422	82.08
SRR4380206	stem base	10DAG_rep3	oligo-dT	61531385	49888709	81.08
SRR5054865	whole grain	3DPA_rep1[d]	oligo-dT	32596699	27026153	82.91
SRR5054851	whole grain	3DPA_rep2	oligo-dT	32459730	26604811	81.96
SRR5054864	whole grain	8DPA-rep1	oligo-dT	32613463	27164779	83.29
SRR5054856	whole grain	8DPA-rep2	oligo-dT	32609773	27086570	83.06
SRR5054854	whole grain	13DPA_rep2	oligo-dT	32599620	24361858	74.73
SRR5054857	whole grain	13DPA_rep1	oligo-dT	32543237	24053873	73.91
SRR5054855	whole grain	18DPA_rep1	oligo-dT	32564906	21544228	66.16
SRR5054863	whole grain	18DPA_rep2	oligo-dT	32555771	21992007	67.55
			**total**	**1474501022**	**1175884967**	**79.74**

[a]SD short day conditions
[b]LD long day conditions
[c]DAG days after germination
[d]DPA days post anthesis
